# Effective surgical management of gastric outlet obstruction symptoms caused by annular pancreas in an adult female: A case report

**DOI:** 10.1016/j.ijscr.2024.110077

**Published:** 2024-07-23

**Authors:** Ali Alshiekh, M Fadi Alkurdi, Rana Hadakie, Mohammed Alarsan, Luna Sukkar, Hamoud Hamed

**Affiliations:** a Department of Surgery, Faculty of Medicine, Damascus University, Damascus, Syrian Arab Republic; b Department of Surgery, Al-Assad University Hospital, Damascus University, Damascus, Syrian Arab Republic; c Department of Biochemistry and Microbiology, Faculty of Pharmacy, Damascus University, Damascus, Syrian Arab Republic; d Department of Gastroenterology and Hepatology, Faculty of Medicine, Damascus University, Damascus, Syrian Arab Republic

**Keywords:** Annular pancreas, Duodenoduodenostomy, Gastric outlet obstruction, Case report

## Abstract

**Introduction:**

Annular pancreas (AP) is a rare condition that usually is not associated with symptoms in adults. However, in some patients, AP may cause non-specific symptoms such as abdominal pain and vomiting, making its diagnosis challenging. The current case report presents a challenging diagnosis of an AP case and surgical management of it by performing duodenoduodenostomy.

**Case presentation:**

A 47-year-old female presented with chronic abdominal pain and vomiting after meals. The examination using CT showed a complete ring of pancreatic tissue encircling the descending part of the duodenum, confirming the diagnosis of AP. Therefore, the patient underwent duodenoduodenostomy, in which the obstruction was bypassed.

**Clinical discussion:**

AP is a rare condition characterized by a band of pancreatic tissue that encircles the second part of the duodenum. Most cases of AP in adults remain asymptomatic. However, when AP is symptomatic, it is associated with vague abdominal symptoms. The primary management of symptomatic AP in adults involves surgical bypass of the annulus through performing gastrojejunostomy or duodenojejunostomy. While duodenoduodenostomy is less favorable, we opted for it due to the limitation of the obstruction to a specific segment of the duodenum.

**Conclusion:**

This case underscores the importance of considering AP as a potential cause in the differential diagnosis of vague and persistent gastrointestinal symptoms. Moreover, most studies concerning the management of AP have consisted of case reports or small case series. This emphasizes the need for further studies to enhance our understanding of the most appropriate approach for managing each case of AP.

## Highlights

• Annular pancreas (AP) is a rare congenital anomaly that is not usually associated with symptoms in adults.
• In symptomatic cases, AP causes non-specific symptoms such as abdominal pain.
• AP is diagnosed by laparotomy or radiologic studies such as CT in adults.
• Duodenoduodenostomy may be an effective approach in managing of AP in adults.

## 1 Background

Annular pancreas (AP) is a rare congenital anomaly that rarely causes symptoms in the adult population. It occurs when a band of pancreatic tissue surrounds the second part of the duodenum, and this surrounding can be either complete or partial [1,2]. When AP is symptomatic, it causes nonspecific symptoms and is incidentally diagnosed during radiological tests conducted to evaluate vague abdominal symptoms. The primary management approach involves surgical intervention, such as duodenojejunostomy or gastrojejunostomy [1]. Herein, we report a case of an adult female who presented to our university hospital with chronic symptoms of gastric outlet obstruction. Based on the findings from CT scan, she was diagnosed with complete AP. Consequently, she underwent duodenoduodenostomy to bypass the obstruction. This work was reported in accordance with the 2023 SCARE criteria [3].

## 2 Case presentation

A 47-year-old female was admitted to our clinic with a main complaint of persisting unexplained abdominal pain for the past 2 years and recurrent episodes of vomiting for the past 6 months. The vomiting was postprandial and non-projectile, while the abdominal pain was mild in intensity and relieved by vomiting.

The patient had no significant medical history except for a cesarean section 11 years ago. Upon clinical examination, there were no remarkable findings except for some weight loss. Additionally, blood tests and abdominal ultrasound were normal.

The patient underwent an upper gastrointestinal endoscopy which revealed a significant amount of fluid and food residue in the stomach, despite the patient fasting properly for the endoscopy. It also showed dilation in the first segment of the duodenum and a narrowed area at the beginning of the second segment (D2) that suggested extrinsic compression. Additionally, an upper gastrointestinal series (barium meal) was conducted, which confirmed the presence of a dilated stomach and a large first segment in the duodenum (D1), measuring 85*105 mm, with narrowing at the end of the dilated area.

**Fig. 1:**
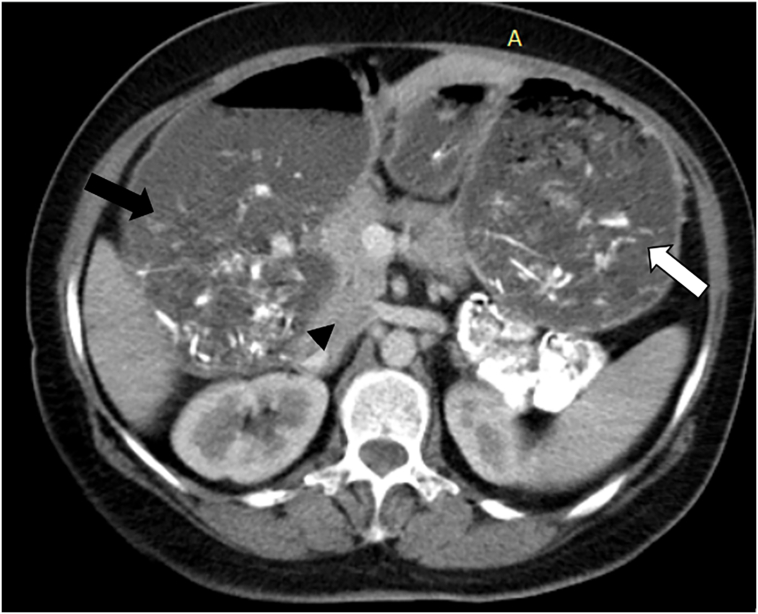
Contrast-enhanced CT images (axial plane) showing a dilation in stomach (white arrow), a dilation of the first part of the duodenum (black arrow) and the head of pancreas (arrowhead).

Further diagnostic tests through performing abdominal CT showed upper gastrointestinal obstruction features without dilation in the second and third parts of the duodenum, which did not align with superior mesenteric artery syndrome. Furthermore, the CT results revealed the presence of pancreatic tissue encircling the second part of the duodenum (Fig. 1) and ruled out malignant lesions of the pancreatic head as the CT scan showed a circumferential appearance of the pancreatic tissue surrounding the duodenum, without any discrete or focal mass in the pancreatic head region. Additionally, there was an absence of pancreatic duct dilatation. There was also a lack of infiltration or invasion into surrounding structures, such as the duodenum or bile duct. Furthermore, there was no evidence of metastatic disease on the CT scan.

**Fig. 2:**
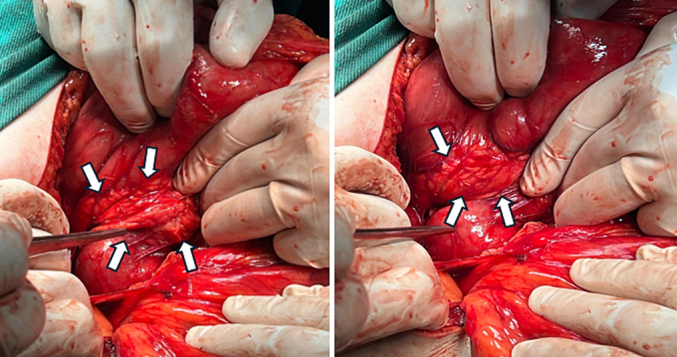
Intraoperative photo of the pancreatic tissue encircling the second part of the duodenum and causing gastric outlet obstruction.

Subsequently, an exploratory laparotomy was performed, in which the existence of a ring of pancreatic tissue that totally encircles the descending part of the duodenum was confirmed. Thus, the diagnosis of annular pancreas was established (Fig. 2). Consequently, a side-to-side duodenoduodenostomy was performed after dissecting the attachment of the duodenum to allow mobilization of the duodenum. Post-operation, the patient was in good health. Therefore, she was discharged on the 6th day of admission, and she reported tolerating a regular oral diet without experiencing any complications.

## 3 Discussion

Annular pancreas (AP) is a rare congenital anomaly in which a ring of pancreatic tissue partially or completely encircles the descending part of the duodenum. It was first described by Tiedemann in 1818 and given its name by Ecker in 1862 [2,4,5]. During embryogenesis, the pancreas starts to develop during the fifth gestational week from two ventral buds that rapidly unite, and a dorsal bud. By the seventh week of gestation, the ventral bud rotates with the duodenum and fuses with the dorsal bud. The ventral bud gives rise to the inferior head of the pancreas and the inferior part of the uncinate process, while the dorsal bud forms the tail and body of the pancreas [1,2,4,6,7]. There are two prominent theories that have been proposed to explain the development of annular pancreas. Lecco's theory proposes that the right ventral bud adheres to the duodenal wall and becomes stretched and elongated after it rotates, resulting in encirclement of the duodenum. Baldwin's theory proposes that the left ventral bud persists and migrates around the duodenum in opposite directions, then fuses with the dorsal bud, encircling the duodenum [8].

AP was previously considered a disease primarily affecting infants and often associated with other congenital anomalies, such as Down syndrome, intestinal malrotation, and cardiac anomalies [5,9]. However, it has been reported that the incidence rate of AP is nearly the same in adults and children. It was estimated that the incidence rate of AP in adults ranges between 0.005 % and 0.015 % [2,9].

Approximately half to two-thirds of adult patients with AP remain asymptomatic. However, when AP becomes symptomatic, abdominal pain, vomiting, postprandial fullness, and pancreatitis are among the most commonly reported symptoms. Moreover, AP may be associated with other symptoms such as upper gastrointestinal bleeding, jaundice, and even neoplasia. These symptoms are often reported between the ages of twenties and sixties [1,2,4,9,10]. Our patient has been experiencing persistent abdominal pain for two years, and in the last six months, this pain has been accompanied by postprandial vomiting. These symptoms may indicate the progression of duodenal obstruction, which could be attributed to recurrent pancreatitis causing scarring [1,11].

While the presence of a double bubble sign in a plain abdominal radiograph, or findings indicating duodenal obstruction observed during an upper gastrointestinal series, may suggest AP in infants, a definitive diagnosis of AP is confirmed through laparotomy [1,5]. In adults, the preferred diagnostic tools for AP are endoscopic retrograde cholangiopancreatography (ERCP) or laparotomy [4,12]. However, radiologic studies, such as computed tomography and magnetic resonance imaging (MRI), can also be used to diagnose AP in adults [1,4,10]. The morphological features of AP may include a configuration of pancreatic tissue resembling a crocodile jaw in cases of incomplete AP, or the presence of a complete ring of pancreatic tissue surrounding the second part of the duodenum, which can appear as a circular, triangular, or sandwich sign [4,6]. However, laparotomy is required to confirm the diagnosis of AP in 40 % of cases [12]. Additionally, other diagnostic tests, such as the findings from an upper gastrointestinal series, may provide suggestive evidence of AP [1]. In relation to biochemical and genetic tests, there is currently no available test specifically for diagnosing AP [12].

The management of AP in patients with symptomatic duodenal obstruction primarily involves surgical bypass which aims to alleviate gastric outlet or duodenal obstruction by bypassing the annulus through the performance of either gastrojejunostomy or duodenojejunostomy in adults. While duodenoduodenostomy can be used for this purpose in adults, it is less favorable due to the decreased mobility of the duodenum compared to infants [1]. However, in our case, we chose duodenoduodenostomy due to the limitation of the obstruction to a specific segment of the duodenum without involving the jejunum, and the possibility of totally bypassing the obstruction through duodenoduodenostomy in this case. This procedure was effective in completely relieving symptoms related to obstruction after surgery. A similar procedure was performed by de la Rosa Rodriguez et al. and Mittal et al., who performed duodenoduodenostomy to manage a complete AP case and a partial AP case in adults, respectively [6,12]. Resection of the annulus is not recommended as it is associated with partial relief of obstruction, fistula formation, pancreatitis, or duodenal stenosis [1,12].

## 4 Conclusion

AP is a rare condition in adults that should be considered when evaluating nonspecific abdominal symptoms. Duodenojejunostomy is the recommended surgical approach for managing AP in adults. However, in our case, we opted for duodenoduodenostomy, which successfully alleviated the symptoms of gastric outlet obstruction associated with AP. The choice of the most suitable procedure for managing symptomatic AP should be based on factors specific to each individual case, including the severity of the obstruction and the surgeon's expertise.

## Abbreviations

AP: Annular pancreas
ERCP: Endoscopic retrograde cholangiopancreatography
CT: Computed tomography
MRI: Magnetic resonance imaging

## Consent for publication

The patient has provided a written informed consent for publication of the case. A copy of the written consent is available for review by the Editor-in-Chief of this journal on request.

## Ethical approval

The ethical committee approval was not required given the article type (case report).

## Funding

This research did not receive any specific grant from funding agencies in the public, commercial, or not-for-profit sectors.

## Research registration number

N/A.

## Guarantor

Ali Alshiekh.

## CRediT authorship contribution statement

**Ali Alshiekh**: General surgeon participated in the operation, Writing – review & editing of the manuscript, and Project administration. **M Fadi Alkurdi**: General surgeon participated in the operation, review of the manuscript. **Rana Hadakie**: Writing – review & editing of the manuscript, Resources, and Project administration. **Mohammed Alarsan**: General surgeon participated in the operation, Writing – review & editing, pictures preparation. **Luna Sukkar**: drafting the manuscript. **Hamoud Hamed**: General surgeon led the surgical procedure, Supervision, review & editing of the manuscript. Manuscript has been read and approved by all named authors.

## Declaration of competing interest

We have no competing interests to declare.

## Data Availability

Not applicable since no datasets were analyzed.

## References

[1] Thukral C., Freedman S.D. (Mar 2024). UpToDate.

[2] Sandrasegaran K., Patel A., Fogel E.L., Zyromski N.J., Pitt H.A. (2009). Annular pancreas in adults. AJR Am. J. Roentgenol..

[3] Sohrabi C., Mathew G., Maria N., Kerwan A., Franchi T., Agha R.A. (2023). The SCARE 2023 guideline: updating consensus Surgical CAse REport (SCARE) guidelines. Int. J. Surg..

[4] Zhou Y., Li X. (2022). Investigation of annular pancreas through multiple detector spiral CT (MDCT) and MRI. J. Appl. Clin. Med. Phys..

[5] Ali Almoamin H.H., Kadhem S.H., Saleh A.M. (2022). Annular pancreas in neonates; Case series and review of literatures. Afr. J. Paediatr. Surg..

[6] Mittal S., Jindal G., Mittal A., Singal R., Singal S. (2016). Partial annular pancreas. Proc. (Baylor Univ. Med. Cent.).

[7] V S.K., Sangu P., C K., R P., Chidambaranathan S., Obla Lakshmanamoorthy N.B. (2022). Congenital anomalies of the pancreas: various clinical manifestations and their impact on pancreatic diseases and outcomes. Cureus.

[8] Lee N.K., Kim S., Jeon T.Y., Kim H.S., Kim D.H., Seo H.I. (2010). Complications of congenital and developmental abnormalities of the gastrointestinal tract in adolescents and adults: evaluation with multimodality imaging. Radiographics.

[9] Zyromski NJ, Sandoval JA, Pitt HA, Ladd AP, Fogel EL, Mattar WE, et al. Annular pancreas: dramatic differences between children and adults. J. Am. Coll. Surg. 2008;206(5):1019–25; discussion 25–7. 10.1016/j.jamcollsurg.2007.12.009 18471747

[10] Alahmadi R., Almuhammadi S. (2014). Annular pancreas: a cause of gastric outlet obstruction in a 20-year-old patient. Am J Case Rep..

[11] Zheng H.M., Cai X.J., Shen L.G., Finley R. (2007). Surgical treatment of annular pancreas in adults: a report. Chin. Med. J..

[12] de la Rosa Rodriguez R., Fogarty A., Israel G.M., Sanchez M.J. (2019). Annular pancreas in a 24-year-old woman with persistent abdominal pain. BMJ Case Rep..

